# Potential role of exosomes derived from the tumor microenvironment in tumor progression and treatment

**DOI:** 10.3389/fonc.2025.1583295

**Published:** 2026-02-06

**Authors:** Ying Yu, Wenjun Zhang, Kun Wang

**Affiliations:** 1 Department of Urology, Zhejiang Provincial People’s Hospital Bijie Hospital, Bijie, Guizhou, China; 2 Department of Urology, Anshun People’s Hospital, Anshun, Guizhou, China

**Keywords:** exosome, tumor microenvironment, pre-metastatic microenvironment, progression, treatment

## Abstract

Exosomes are important intercellular communication substances that connect the intercellular communication between the tumor microenvironment and the premetastatic microenvironment during tumor progression. By carrying different regulatory substances, such as proteins, cytokines, nucleic acids, etc., to regulate tumor progression, including angiogenesis, invasion, metastasis, drug resistance and other aspects. In addition to their potential as diagnostic markers, exosomes can also be used as excellent drug delivery carriers. Due to their excellent targeting and histocompatibility, exosomes have considerable application prospects in tumor therapy. This reviews recent studies on tumor-related exosomes, summarizes the mechanisms of intercellular communication and regulation of exosomes in tumor microenvironment and pre-metastasis microenvironment, and summarizes the current research progress on exosomes in tumor therapy.

## 1 Exosome; tumor microenvironment; pre-metastatic microenvironment

Cancer remains a formidable global health challenge. Despite advances in diagnosis and therapy, metastasis, recurrence, and treatment resistance continue to be the primary obstacles to improving patient survival. These challenges persist because a tumor is not simply a mass of cancer cells but a complex “organ” whose development and behavior are critically shaped by its surrounding tumor microenvironment (TME).The critical importance of the TME was first highlighted by Stephen Paget’s 1889 “seed and soil” hypothesis, which proposed that metastatic cancer cells (“seeds”) can only proliferate within a supportive organ-specific microenvironment (“soil”). While this theory established the TME’s importance, research has historically focused on the unidirectional influence of cancer cells on their surroundings—the “seed to soil” axis. We recognize that this crosstalk is highly dynamic and bidirectional. A pivotal shift in perspective acknowledges that the “soil” is not passive; it actively instructs cancer cells, influencing their fate through “soil to seed” signaling. This paradigm elevates the TME to a dominant driver of tumor progression. Among the various mediators of this intercellular communication, exosomes have emerged as key messengers in this “soil to seed” dialogue.

**Figure 1:**
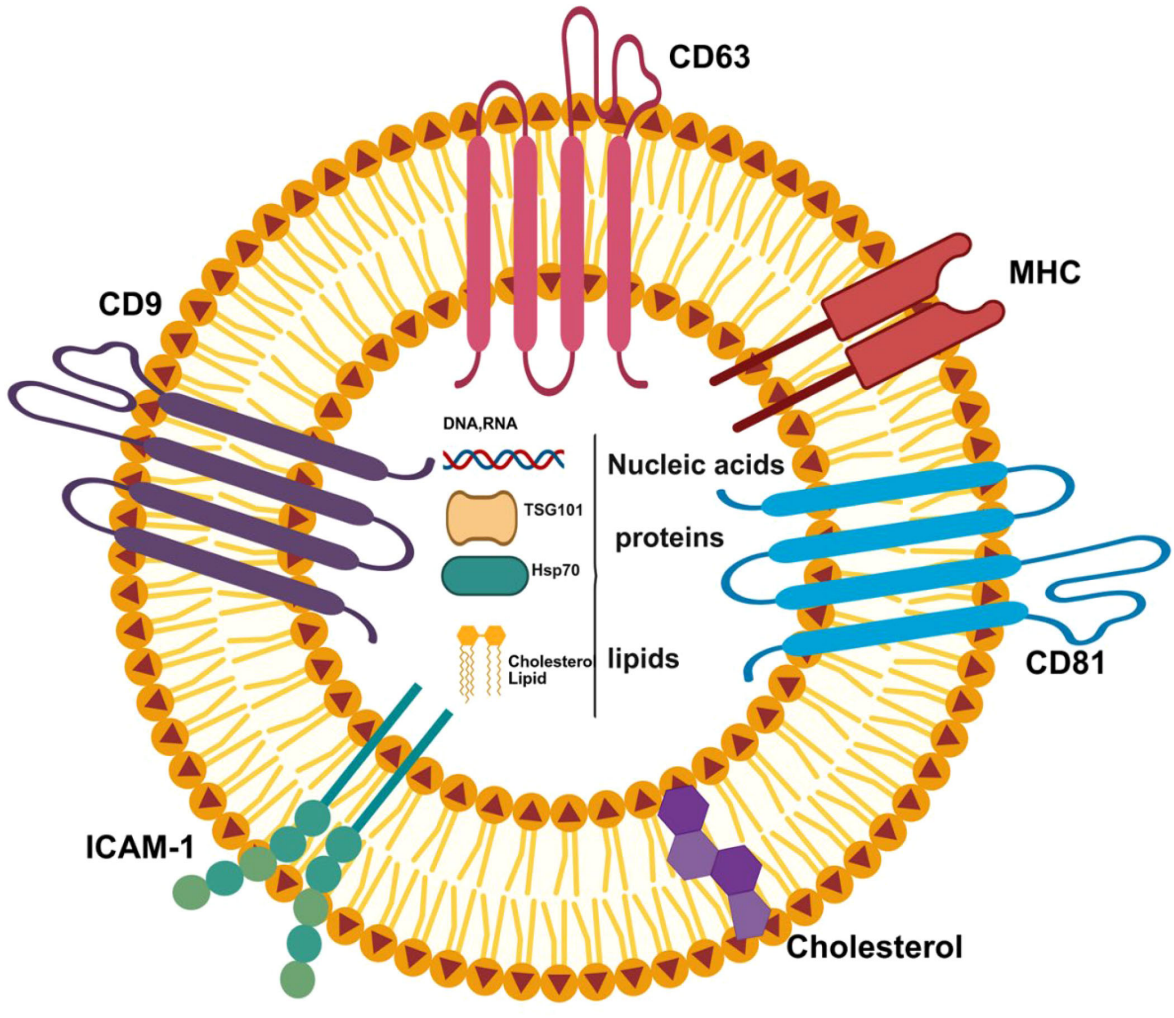
The structure of exosomes:Exosomes are mainly composed of lipids, functional proteins (such as membrane proteins, cytoplasmic and nuclear proteins), extracellular matrix proteins, and nucleic acids (fragments of DNA and RNA).

Extracellular vesicles (EVs) are tiny structures derived from cell membranes and are often called microvesicles or nanovesicles. All prokaryotic and eukaryotic cells can secrete EVs in an evolutionarily conservative manner. Although early views were that EVs were simply waste products or damaged products of cellular metabolism, subsequent research revealed their key biological functions and established their status as important cellular components. EVs can be divided into several types according to their size, biological origin, and location of formation within cells. Common ones include exosomes, microparticles, exfoliative vesicles, apoptotic bodies, etc. There are two main pathways for its biogenesis: one is direct sprouting of the cell membrane; the other involves the endocytotic system, which is released through the fusion of multivesicles and the plasma membrane. Exosomes are important communication substances between cells. (The description of exosomes is illustrated in **Figure 1**). The polyvesicles formed from the intracellular body are released into the extracellular environment after fusion with the plasma membrane, and the diameter of the vesicles is 30~150nm. Exosomes have lipid bilayers that can carry a variety of regulatory substances, including proteins, lipids, and nucleic acids (1–5). The production of exosomes is regulated by a variety of factors. Current studies have confirmed that the protein network closely related to the formation of exosomes involves Rab GTPase proteins, quadritransmembrane proteins, such as CD9, CD81 and CD63, and lipid modifying enzymes, such as sphingomatase. And the interaction of endosomal sorting complexes required for transport (ESCRT). However, the specific sorting mechanism of exosome-carried cargo has not yet been clarified (2, 6). Exosomes are widely distributed in the body and have been detected in tissue fluids such as milk, cerebrospinal fluid, blood, lymph fluid, semen and prostate fluid, and have shown potential as diagnostic and therapeutic markers in the field of liquid biopsy of tumors (7, 8). Exosomes have excellent targeting ability and penetrating power, and exosomes constructed by engineering also have great development potential in the current field of tumor therapy (9).

The tumor microenvironment contains a variety of cell populations, such as fat cells, cancer-associated fibroblasts, mesenchymal stem cells, endothelial cells, and immune cells (10). The communication between tumor cells and different cell populations in the tumor microenvironment regulates tumor progression and enables tumor cells to constantly adapt to the new living environment (10, 11). Tumor-derived exosomes are more active intercellular communication substances during tumor progression and participate in swelling. The construction of the tumor microenvironment and the pre-metastatic niche can make the tumor show resistance to the changing environmental pressure by reshaping the tumor microenvironment during the process of tumor progression. Moreover, regulatory substances carried by exosomes derived from other constituent cell populations in the microenvironment can also act on tumor cells, breeding more aggressive and heterogeneous tumor cell phenotypes (11, 12).

This review will be based on the emerging paradigm of “soil to seed” and systematically elaborate on how exosomes derived from various stromal cells in the tumor microenvironment can serve as key communication media. In view of the in-depth research on tumor-derived exosomes in recent years, this reviews the previous literature, mainly focusing on the cell-to-cell interaction mechanisms of exosomes in the construction of the tumor microenvironment and tumor pre-metastasis niche during tumor progression, as well as exosome-mediated tumor therapy.

## 2 Exosomal communication between cancer-associated fibroblasts and tumor cells

**Figure 2:**
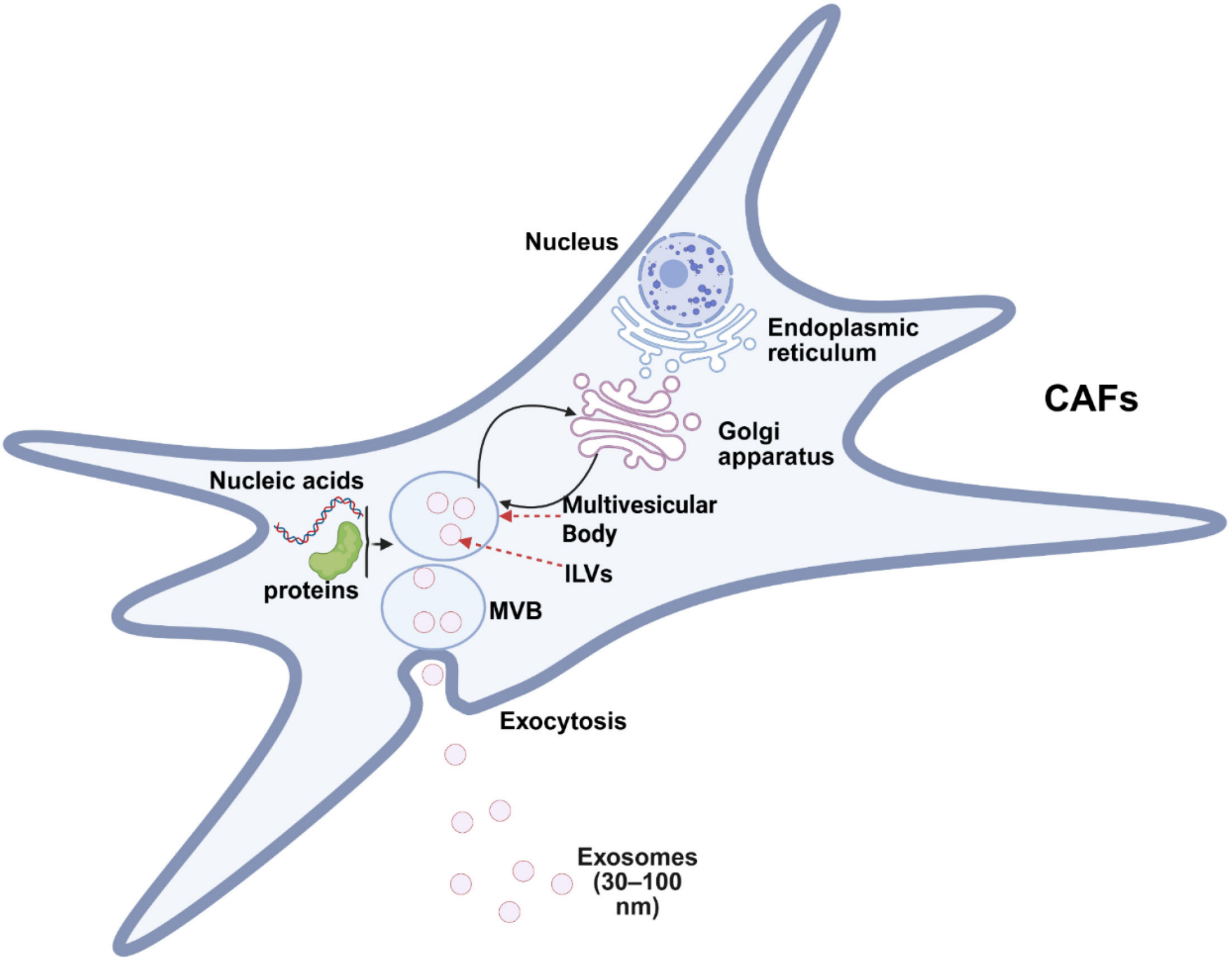
Exosome communication between cancer-associated fibroblasts and tumor cells: The process of exosome formation: a) Inward invagination of the cell membrane and early endosomes; b) Formation of multivesicular bodies containing intraluminal vesicles through the sorting and transport pathway of early endosomes; c) Eventual fusion of the multivesicular bodies with the plasma membrane, and secretion of the intraluminal vesicles within them as exosomes.

Under normal conditions, fibroblasts maintain a low level of cell proliferation and metabolic activity and are activated during tissue injury or inflammatory responses (13). Cancer-associated fibroblasts (CAF) detected during tumor progression are a heterogeneous fibroblast type and one of the important cell populations constituting the tumor microenvironment (10, 13). Some of these subtypes have cancer-inhibiting effects, while others have the effect of promoting tumor progression (14, 15). Tumor-associated fibroblasts influence tumor phenotypes through contact with tumor cells or secretion of cytokines and exosomes, and play an important role in cancer cell invasion and metastasis, immune escape, angiogenesis and drug resistance (The exosome communication between cancer-associated fibroblasts and tumor cells is shown in **Figure 2**.) (16, 17).

**Figure 3:**
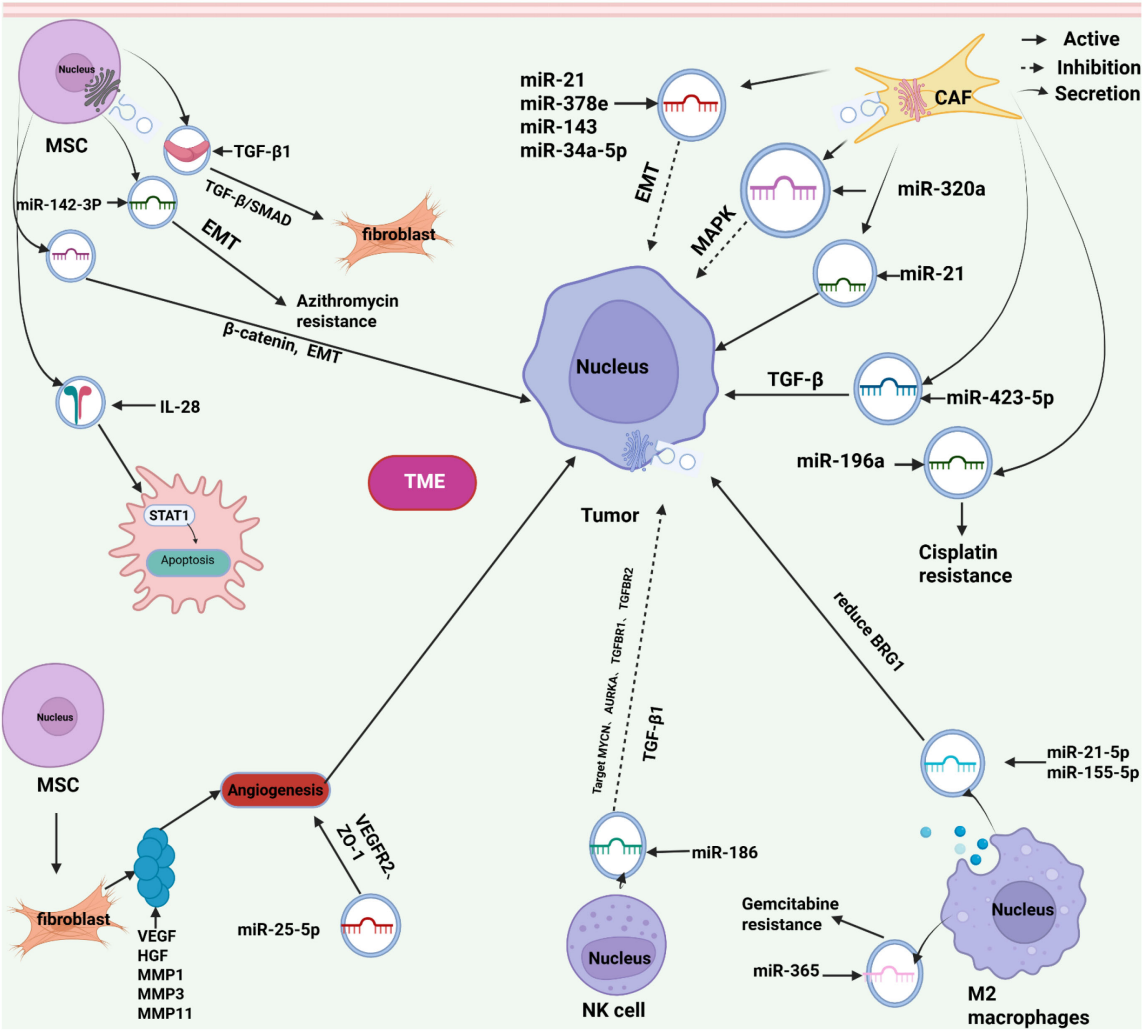
Exosome communication between various cells in the tumor microenvironment and tumor cells: Tumor-associated cells further promote tumor proliferation, migration, and metastasis by secreting cytokines and exosomes and regulating corresponding signaling pathways. Mesenchymal stem cells enhance tumor resistance through paracrine mechanisms such as cytokines and exosomes, which induce epithelial-mesenchymal transition and apoptosis. The signals delivered by extracellular vesicles from tumor cells can recruit and induce the reprogramming of immune cells (including various related cells) infiltrating the tumor, thereby exerting immunosuppressive activity and promoting the biological progression of the tumor.

Previous studies have confirmed that cancer cells induce a variety of cell types to differentiate into cancer-related fibroblasts through exosome communication, and participate in extracellular matrix remodeling and tumor microenvironment reprogramming to promote tumor progression (18). In a cell experiment, it was confirmed that tumor-associated fibroblasts not only promoted metabolic reprogramming of prostate and pancreatic cancer cell lines, inhibited mitochondrial oxidative phosphorylation, and increased glycolysis and glutamine-dependent reduction carboxylation in cancer cells. It can also be used as an energy supply bank to supply amino acids to energy-deficient tumor cells, so that tumor cells can continue to grow in the case of hypoxia and reduced nutrient sources (19). In a study of patients with liver cancer, levels of the exosome miR-320a derived from tumor-associated fibroblasts were significantly reduced As shown in **Figure 3**. The authors demonstrated that miR-320a could inhibit the proliferation, migration and metastasis of hepatoma cells by binding PBX3 and inhibiting the activation of MAPK pathway as shown in **Table 1**. It has been further confirmed in animal experiments that overexpression of miR-320a can inhibit tumor progression (20). In another study on breast cancer, increased expression levels of miR-21, miR-378e, and miR-143 were confirmed in exosomes of tumor-associated fibroblasts. Uptake of exosomal miRNA by cancer cells can enhance cell dryness and induce epithelial mesenchymal transformation (EMT) (21). The study of Bhome et al. also found that the enrichment of miR-21 in exosomes derived from tumor-associated fibroblasts would promote liver metastasis of colon cancer (26). In addition, oral squamous cells. It has been confirmed in cancer cell experiments that the tumor-associated fibroblast derived exosome miR-34a-5p regulates the epithelial-mesenchymal transformation of tumor cells (27).

**Table 1:** Specific exosomes in tumor progression.

Specific exosomes during tumor progression				
Source of exosomes	The key molecules carried	Main Function	The impact on tumor progression	References
CAFs	miR-320a	Inhibit the MAPK pathway	Inhibit the proliferation, migration and metastasis of cancer cells	(20)
	miR-21, miR-378 and miR-143	Induction of epithelial mesenchymal tmnsition (EMT)	Promote the proliferation, migration and metastasis of tumor cells	(21)
MSCs	miR-142-3p	Inhibiting and activating NOTCHI to induce epithelial-mesenchymal transition (EMT)	Enhance the chemoresistance of tumors to doxorubicin	(22)
Tumor cells under hypoxia	miR-105	Reduce the expression of ZO-I	Promote the entry of tumor cells into the circulation	(23)
Tumor vascularization microenvironment	miR-25-3p	Down-regulate the expression of the tight junction protein ZO-1 in endothelial cells	Promote tumor metastasis	(24)
Immune cell	miR-21-5p and miR-155-5p	Down-regulate the expression of the gene BRG1	Promote the invasion and migration of cancer cells	(25)

In the study on tumor drug resistance, Yeung et al. found that the exosome miR-21 derived from tumor-associated fibroblasts could bind to APAF1 after metastasis to ovarian cancer cells, inhibit tumor cell apoptosis and enhance paclitaxel resistance (28). Cancer-associated fibroblasts can deliver miR-196a to tumor cells via exosomes to increase cisplatin resistance in advanced head and neck cancer cells (29). In another study, it was confirmed that tumor-associated fibroblasts regulate the TGF-β pathway through the exosome miR-423-5p, thereby promoting the resistance of prostate cancer cells to taxane treatment (30). In addition, lncRNA was also detected in exosomes derived from tumor-associated fibroblasts. In studies on colorectal tumors, it was found that tumor-associated fibroblasts transferred H19 to tumor cells through exosomes, and H19 could act as a miRNA sponge to bind miR-141 in cancer cells, thereby activating the β-catenin pathway. Enhance the dryness and drug resistance of cancer cells (31).

## 3 Exosomal communication between mesenchymal stem cells and tumor cells

Mesenchymal stem cells (mesenchymal stem cells) are pluripotent stem cells widely present in human tissues, originating from the mesoderm and neural crest, and capable of differentiating into multiple lineages of mesoderm cells, such as osteoblasts, adipocytes, chondrocytes, endoderm and neuroectoderm cells under the stimulation of the surrounding environment (32, 33). Mesenchymal stem cells have strong plasticity and participate in the maintenance and regulation of various stem cell ecological niches in tissues, such as angiogenesis, bone regeneration, hematopoietic and immune regulation, through paracrine, such as cytokines and exosomes, in the interaction with the surrounding environment (32). As the hub of stem cell niche regulation in tissues, impaired proliferation, differentiation bias and dysfunction of mesenchymal stem cells have been proven to be the basis of tissue changes in aging and various diseases (33, 34). In the process of tumor progression, the central function of mesenchymal stem cells in maintaining the stem cell niche is well utilized by cancer cells, which can recruit mesenchymal stem cells in the primary and metastatic sites and induce mesenchymal stem cells to build a microenvironment conducive to tumor growth, further enhance the stemness of cancer cells, and participate in the induction of more invasive tumor heterogeneity phenotype. Assist the tumor to better adapt to the pressure of the surrounding environment (35–37).

Postmortem examination of castration-resistant prostate cancer patients showed that mesenchymal stem cells were abundant in both primary and metastatic sites, and had the potential to differentiate into adipose, osteoblast, and chondrocyte. Moreover, through comparison, researchers found that the function of mesenchymal stem cells was often inhibited in samples of prostate hyperplasia, while the function of mesenchymal stem cells was active in prostate cancer tissues, and their count in tumor tissues was related to the prognosis of prostate cancer (38). Another study found that exosomes from cancer cells are rich in TGFβ1, which can induce bone marrow sources, adipose tissue sources, and umbilical cord by activating the TGFβ/SMAD pathway.

The derived mesenchymal stem cells differentiate into fibroblasts (10). In the study of the effect of mesenchymal stem cells on tumor invasion and drug resistance, researchers found that bone marrow-derived mesenchymal stem cells, via exosomes, deliver miR-142-3p to colon cancer cells, induce tumor epithelial mesenchymal transformation (EMT) and enhance tumor chemical resistance to adriamycin by inhibiting NUMB and activating NOTCH1 (22). Another study found that exosomes of adipose-derived mesenchymal stem cells can positively regulate the β-catenin pathway, promote epithelial mesenchymal transformation of breast cancer cells, and promote tumor invasion and metastasis (39). In a recent study, the researchers found that at the site of prostate cancer bone metastasis, tumor cells recruit fibroblast stem cells and induce their differentiation into osteoblasts to participate in the construction of tumor microenvironment. But mesenchymal stem cells secrete interleukin-28(IL28) induces apoptosis of prostate cancer cells by activating the STAT1 pathway of tumor cells. Prostate cancer cells left over from long-term screening were resistant to interleukin-28 induced apoptosis and also showed resistance to docetaxel (40).

## 4 Exosome communication in the tumor angiogenic microenvironment

With the continuous growth of tumors, hypoxia and nutrient deficiency within tumors become more significant, and angiogenesis becomes active in the tumor microenvironment (41). The establishment of more blood vessel networks not only improves the tumor microenvironment lacking in oxygen and nutrients, but also further promotes tumor invasion and metastasis through intercellular communication (41–43). During this process, tumor cells secrete vascular endothelial growth factor (VEGF), fibroblast growth factor (FGF), platelet-derived growth factor (PDGF), and basic fibroblast growth factor (bFGF), transforming growth factor β (TGF-β), tumor necrosis factor α (TNF-α) and interleukin-8 (IL-8) exosomes, induce capillary budding. Further, the vascular endothelial cells at the top of germination secreted high levels of delta-like -4 protein (DL-4), which activated Notch pathway in adjacent microvascular endothelial cells and further induced capillary germination (12, 42). Mesenchymal stem cells recruited by angiogenesis signals can be induced to differentiate into fibroblasts, and fibroblasts derived from mesenchymal stem cells can assist in the remodeling of the outer matrix of tumor cells by secreting VEGF, hepatocyte growth factor (HGF), MMP1, MMP3 and MMP13, and enhance the invasion and migration ability of tumor cells. Thus promoting tumor progression (44).

Exosomes have been shown to be active intercellular communication substances in the tumor angiogenic microenvironment. Studies have confirmed that glioblastoma-derived exosomes contain high levels of miR-221, proteoglycan glypican-1 and syndecan-4, which increase revascularization by enhancing the proliferation and formation of endothelial cells and tubules (45). In addition, exosomes from head and neck squamous cell cancer cell lines are reprogrammed and mediated by endothelial cells regulation induces angiogenesis (46).

In addition, compared with normal blood vessels in tissues, the vascular network constructed inside tumors is more loosely connected between cells, which is more conducive for tumor cells to enter the circulatory system for further dissemination. Evidence from previous studies in the hypoxic tumor microenvironment, breast cancer cells will secrete more exosomes, among which, in addition to inducing angiogenesis, exosome miR-105 can down-regulate the expression of endothelial cell tight junction protein ZO-1, thereby increasing vascular permeability and promoting tumor cells to enter the circulation (23). Another study showed that colorectal cancer cell-derived exosomes miR-25-3p regulate the expression of VEGFR2, ZO-1, Occludin and Claudin5 in endothelial cells by targeting KLF2 and KLF4 to promote vascular leakage and promote tumor metastasis. Further animal experiments have shown that this regulatory effect can be extended to tumor metastasis and participate in the construction of tumor microenvironment before metastasis (24).

## 5 Exosome communication between GRP78 and tumor cells

The detection of exosomal proteins is extremely challenging, which makes it particularly important to understand the mechanism by which they promote cancer stemness. GRP78 is a membrane protein of the HSP70 family. Besides participating in protein quality control and UPR responses as an endoplasmic reticulum chaperone, it can also initiate multiple signaling pathways on the cell membrane. Since its discovery in hamster cells in 1984, its high expression in cancer cells has been confirmed to drive tumor malignancy progression by promoting proliferation, survival, invasion, and metastasis (4, 5).

Studies have shown that through GRP78-induced macrophage exosomes, miR-769-5p is delivered to colorectal cancer cells. The GRP78-induced macrophage exosomes promote the induction of chemotherapy resistance in CRC cells. A study based on the release of GRP78 by tumor cells into the microenvironment through exosomes pointed out that traditional Chinese medicine salviic acid A can interact with SGRP78, confirming that this finding may play a potential anti-angiogenic role in the tumor microenvironment. Recently, a study on gastric cancer has been confirmed. The exosomes rich in GRP78 have a promoting angiogenesis function and can significantly enhance the activity of vascular endothelial cells. Furthermore, studies have revealed a new mechanism by which the traditional Chinese medicine Fisetin induces cell apoptosis and endoplasmic reticulum stress, which is achieved by promoting the generation of GRP78 exosomes (47–52).

## 6 Exosome communication between immune cells and tumor cells

The tumor microenvironment is one of the important and complex components of the microenvironment in the process of tumor progression. The tumor microenvironment is characterized by hypoxia, acidity and nutrient deficiency, which damages the function of immune cells such as NK cells and dendritic cells in most tumor microenvironments, and then accumulates and activates more immunosuppressive cell subsets such as bone marrow-derived suppressor cells and regulatory T cells. And promote tumor immune escape (53, 54). Numerous studies have confirmed that the signals delivered by tumor cells through extracellular vesicles have been shown to recruit and induce the reprogramming of infiltrating immune cells in tumors to exert immunosuppressive activities and promote the progression of tumors (55, 56). This exosome-mediated tumor-immune cell communication involves numerous immune cells, such as regulatory B (Breg) cells, regulatory T (Treg) cells, bone marrow-derived suppressor cells (MDSC), and M2-like tumor-associated macrophages (TAM) and neutrophil (53, 56). It was confirmed in a study of liver cancer that tumor exosomes, by delivering HMGB1(long non-coding RNA) to regulatory B cells, promote the expansion of Breg cells through toll-like receptor (TLR) 2/4 and mitogen-activated protein kinase (MAPK) signaling pathways to regulate the expression of high levels of IL-10. Strongly inhibits the activity of CD8+T cells and promotes the immune escape of tumors (57). Another study showed that patients with gastric cancer carried more TGF-β1 circulating exosomes than healthy patients, as evidenced by cell experiments it can induce differentiation of naive T cells into regulatory T cells and is associated with lymph node metastasis of tumors (58). In a previous study, in xenografted mouse models, tumor-derived exosomes included soluble substances and exosomes. The former can promote the proliferation of bone marrow-derived suppressor cells by activating the Erk pathway, while the exosome surface protein heat shock protein (HSP72) triggers the activation of Stat3 in the bone marrow-derived suppressor cells in a TLR2/MYD88-dependent manner and produces IL-6 through autocrine, thus promoting its immunosuppressive function (59). In another study on liver cancer, it was confirmed that hematoxylated pyruvate was present in liver cancer cells kinase M2 interacts with ARRDC1, the protein of cell membrane microvesicle germination, and is more sorting into tumor exosomes. When delivered to monocytes, kinase M2 can induce metabolic reprogramming of monocytes and promote differentiation of monocytes into macrophages by inducing STAT3 phosphorylation in cells. The chemokine CCL1 secreted by monocytes can further interact with liver cancer cells to produce more exosomes carrying hematoxylated pyruvate kinase M2, thus promoting the progression of liver cancer. In addition, researchers have found that pyruvate kinase M2 has the potential to be used as a diagnostic marker in liver cancer plasma samples (60). tumor suppression of innate immune cells by exosomes has also been studied. Studies have confirmed that tumor exosomes transfer activated epidermal growth factor receptor (EGFR) to host macrophages, thereby inhibiting innate antiviral immunity (61). Another study found that exosomes from colorectal cancer can carry Fas ligands and tumor necrosis factor-associated apoptosis-inducing ligands (TRAIL) to induce apoptosis of activated CD8 T cells (62). In addition, the surface of exosomes derived from mesothelioma and prostate cancer cells can carry ligands of the natural killer cell surface receptor NKG2D, and target the TGF-β pathway to down-regulate the expression of NKG2D on the surface of natural killer (NK) cells and CD8+ T cell membranes, thus damaging the function of NKG2D-dependent cytotoxic lymphocytes and promoting escape (63). At present, the treatment of tumor-targeting immune checkpoints has shown great potential. Studies have found that PD-L1 carried on the surface of exosomes released by tumor cells can inhibit the function of CD8+T cells and the activation of T cells in lymph nodes, thus promoting tumor immune escape. Peripheral blood exosome PD-L1 can be used as a biomarker to evaluate the efficacy of anti-PD-1. In addition, inhibition of tumor-derived exosomes PD-L1 can produce durable systemic anti-tumor immunity (64, 65).

In addition, exosomes from immune cells have two effects on tumors, namely promotion and inhibition. It has been reported that long non-coding RNA (HISLA) obtained by breast cancer cells from exosomes delivered by tumor-associated macrophages can enhance the glycolysis and chemical resistance of breast cancer cells (66). Another study found that in cell experiments, M2 macrophage-derived exosomes miR-21-5p and miR-155-5p down-regulated the expression of gene BRG1, promoting the invasion and migration of colon cancer cells (25). In addition, an animal experiment of pancreatic cancer found that tumor-associated macrophages could deliver miR-365 to cancer cells through exosomes, thereby up-regulating the triphosphate nucleotide metabolism in cancer cells and inducing cytidine deaminase to weaken and inactivate gemcitabine activation, resulting in drug resistance (67). Conversely, it has been found that exosomes derived from alveolar macrophages regulate STAT-induced inflammatory responses in neighboring epithelial cells and inhibit the proliferation and survival of lung adenocellular cells by delivering an inhibitor of the cytokine SOCS3 to cancer cells (68). In addition, another study found that exosomes from NK cells carrying functional miR-186 can target neuroblastoma or tumor infiltration reducing the expression of MYCN, AURKA, TGFBR1 and TGFBR2 genes in NK cells can reduce tumor growth and TGFβ1-dependent immune escape in high-risk neuroblastoma patients (69).

## 7 Exosomes and the construction of the microenvironment before tumor

Stephen Paget’s 1889 “seed and soil” hypothesis of tumor metastasis was a precursor to tumor metastasis (70). Tumor cells entering the circulation are regarded as the seeds of tumor dissemination, and the appropriate “soil” for tumor metastasis is gradually revealed with the study of the microenvironment before tumor metastasis. Exosomes are one of the important participants in the construction of the microenvironment before tumor metastasis. Studies have confirmed that exosomes can enter the circulatory system through the loose vascular structure inside the tumor, establish communication with cells in distant metastatic niches by carrying regulatory substances, and contribute to the construction of the pre-metastatic microenvironment for tumor metastasis (71, 72). In addition, the expression pattern of exosome surface integrins is different in different tumors, and the differential combination of such integrins is significantly correlated with the organ-specificity of different tumor metastases (71, 73). Among them, Hoshino et al. ‘s study on melanoma-derived exosomes confirmed that the exosomal integrin combination α6β4 and α6β1 were significantly associated with lung metastasis, while the integrin combination αvβ5 was significantly associated with liver metastasis. Targeting integrins α6β4 and αvβ5 can reduce specific uptake of exosomes and reduce lung and liver metastasis in mice (73). In addition, another study showed that prostate cancer cells produce a high abundance of αvβ3 integrin on the surface of exosome membranes and can be detected in the blood circulation, and tumors can transmit integrin signals through exosomes (74). Integrin signaling has been confirmed to participate in extracellular matrix remodeling of prostate cancer cells, increase the migration and invasion ability of prostate cancer, and mediate the bone metastasis of tumors (75).

Exosomes play a role in the construction of lung and liver metastasis niches by establishing communication with immune cells or stromal cells (71). A previous study demonstrated that during the construction of pre-metastatic lung niche, tumor-derived exosomes deliver miRNAs to lung epithelial cells to induce lung chemokine secretion and promote neutrophil recruitment by activating toll-like receptor 3 (TLR3) in lung epithelial cells, thus promoting the construction of pre-metastatic niche (76). In addition, Annexin II carried by exosomes of breast cancer cell origin is significantly associated with lung and brain metastases, and Annexin II transferred by exosomes to lung interstitial cells stimulates the activation of STAT3 and p38MAPK-NF-κB pathways in macrophages. Thus, a large amount of IL6 and TNF-α are secreted, resulting in the formation of a pro-tumor growth environment at the site of lung metastasis (77). During the construction of the microenvironment before tumor metastasis, macrophages in liver tissue play a key role in receiving and transmitting tumor exosome information to promote liver organ metastasis. Exosomes of pancreatic cancer cells are phagocytosed by macrophages through CD36-dependent pathway, and the innate macrophage population in the liver is retained at the site of tumor metastasis by accumulating exosomal regulatory signals continued infiltration promotes the development of liver metastases (78). Another similar study demonstrated that colorectal cancer cell-derived exosomes can be absorbed by macrophages, and that the exosomes carry the toll-like receptor 7 in miR-21 macrophages (TLR7) binds and induces liver macrophages to be polar into pro-inflammatory phenotype, and induces inflammatory infiltration of pre-metastatic liver niche through secretion of IL-6, promoting liver metastasis (79).

Tumor-derived exosomes regulate local stromal cells, osteoclasts, and osteoblasts in bone tissue during the construction of bone metastasis niches. In the study of liver cancer, it has been confirmed that cell lines with high invasive ability can transfer CXCR4 to stem cell carcinoma with low invasive ability through exosomes to increase tumor invasiveness, and can also promote the proliferation of lymphatic endothelial cells and increase the formation of lymphatic vessels through the induction of exosomes CXCR4 (80). Melanoma-derived exosomes carry receptor tyrosine kinases that induce vascular leakage at pre-metastatic sites and reprogram bone marrow progenitor cells to a pro-angiogenic phenotype. By reducing the expression of receptor tyrosine kinase in exosomes, the pro-transfer phenotype of bone marrow cells is decreased (81). Another study on non-small cell lung cancer confirmed that epidermal growth factor receptor ligand Amphiregulin expression was highly abundant on the surface of plasma exosome membrane of patients, which promoted the up-regulation of RANKL through continuous activation of pre-osteoclast EGFR signal, increased the expression of proteolytic enzyme, and promoted the pre-metastatic niche construction of osteolytic phenotype (82). Another study found *in vitro* cell experiments that breast cancer cell-derived exosomes can deliver peroxoreducin 4 (PRDX4) to osteoclasts, inducing osteolysis (83). In addition, exosomal miR-141-3p derived from prostate cancer cells can be delivered to osteoblasts. By decreasing the expression of DLC1, the activity of osteoblasts is promoted and the pre-metastatic niche construction of osteoblastic phenotypes is promoted (84). Another study on prostate cancer and breast cancer cell line derived exosomes confirmed through cell experiments that tumor cell derived exosomes have a high abundance of miR-940, and the absorption of exosomes by human mesenchymal stem cells will induce their differentiation into osteoblasts, thus participating in the construction of tumor bone metastasis niche (85).

## 8 Exosome delivery system and tumor therapy

Exosomes have high histocompatibility, small size, can freely pass through the blood-brain barrier, and have great potential as targeted delivery carriers for tumor drugs (12, 86, 87). Currently, exosomes are used in tumor therapy the field covers chemotherapy, gene therapy, immunotherapy, photothermal therapy and photodynamic therapy, etc. (9, 87, 88) as shown in **Table 2**. In a drug study, researchers used engineered macrophage-derived exosomes to carry paclitaxel for targeted delivery to lung metastases, which could significantly improve the efficacy of anti-tumor drug therapy and reduce drug complications (89). Another study has shown that mesenchymal stem cell-derived exosomes have a role in prostate cancer targeted exosomes derived from let-7c have a high abundance and are delivered to castration-resistant prostate cancer cells the proliferation and migration capacity of cells can be significantly reduced (92). In another study on colon cancer, the delivery of miR-21 to colon cancer cells by engineered exosomes reversed drug resistance in colon cancer (90). In addition, exosomes derived from genetically engineered T cells expressing chimeric antigen receptors (CAR) have also shown potential for tumor therapy, and a recent study has shown that genetically engineered T cell therapies expressing chimeric antigen receptors (CAR) are susceptible to immunosuppressive mechanisms. Exosomes released by CAR-T cells express high levels of cytotoxic molecules and inhibit tumor growth. Compared with CAR T cells, CAR exosomes do not express programmed cell death protein 1 (PD1), and recombinant PD-L1 treatment does not reduce its antitumor effect. After experimental model tests, CAR exosome therapy is safer than CAR-T therapy (93). Another study, by loading the MYC gene-targeting CRISPR/Cas9 system into CAR-T, can quickly and effectively target the MYC oncogene to treat B-lymphocytic leukemia. In photodynamic therapy, the preparation of AIEgen hybrid nanovesicles (DES) by electroporation can significantly promote the efficient penetration of effector substances, and the combination of dexamethasone can significantly enhance the photodynamic therapeutic effect of hybrid nanovesicles and effectively inhibit tumor growth (91, 94).

**Table 2:** Therapeutic effects of exosomes in tumors.

Exosomes in tumor treatment		
Tumor treatment methods	Significance	References
Chemotherapy	Exosomes derived from macrophages carry paclitaxel	(89, 90)
gene therapy	CRISPR/Cas9	(91)
Immunotherapy	CAR exosomes	(9, 87, 88, 91)
Photothermal therapy and Photodynamic	AlE-emitting substances (AlEgens) hybridized nanovesicles (DES)	(91)

## 9 Summary and outlook

In the process of tumor progression, exosomes, as widespread intercellular communication substances, play an important regulatory role in promoting tumor progression through intercellular communication including tumor microenvironment and pre-metastatic microenvironment. How to sort the regulatory substances carried by exosomes is still an open question. Due to the limitations of the current exosome separation and extraction technology, the exosome cannot be completely separated from the tissue. In the future, more efficient and stable exosome extraction technology needs to be developed to better interpret the complex exosome communication network inside the tumor. At the same time, exosomes show excellent targeting and histocompatibility in tumor therapy, and have great development potential in the field of tumor precision therapy, but it is still in the experimental stage, and more extensive and rigorous studies are needed to demonstrate it in the future.

The research on exosomes and the tumor microenvironment is moving from a descriptive stage to a new era of mechanism analysis and precise intervention. Future breakthroughs will heavily rely on technological innovations (achieving single-particle, *in vivo*, multi-omics analysis) and the integration of concepts (deeply integrating exosome biology with immunology, metabolism, microbiology, and bioengineering). Although there are still severe challenges in standardization, traceability, and *in vivo* functional validation, diagnostic tools and treatment platforms targeting exosomes undoubtedly have great potential for clinical translation and are expected to become an important component of future cancer precision medicine.

Based on the latest research, we systematically reviewed the mechanisms by which exosomes regulate the tumor microenvironment and the cell communication in the pre-metastatic microenvironment. Subsequently, focusing on the inherent targeted delivery function of exosomes, we summarized the progress in tumor treatment research using exosomes as drug carriers in order to demonstrate their therapeutic potential and lay the foundation for further exploration in this field. .
